# Advancing minimally invasive surgery: A cutting-edge cable-actuated conveying mechanism for reliable tissue transportation

**DOI:** 10.1371/journal.pone.0295585

**Published:** 2023-12-14

**Authors:** Vera Gesina Kortman, Yinte Verberne, Jovana Jovanova, Aimée Sakes

**Affiliations:** 1 Faculty of 3mE, Department of BioMechanical Engineering, Delft University of Technology, Delft, The Netherlands; 2 Faculty of 3mE, Department of Marine and Transport Technology, Delft University of Technology, Delft, The Netherlands; University of Enna Kore: Universita degli Studi di Enna ’Kore’, ITALY

## Abstract

**Introduction:**

Tissue extraction plays a crucial role in various medical disciplines, with aspiration catheters serving as the prevailing method. Unfortunately, these catheters face limitations such as clogging and dependence on tissue properties and device dimensions. Therefore, there is a pressing need for an improved tissue extraction device that enables efficient and reliable tissue removal during Minimally Invasive Surgery (MIS).

**Methods:**

In this study, we present a novel tissue transport system that utilizes a cylindrical conveyor belt mechanism for reliable tissue transportation. We conducted experiments using a proof-of-principle prototype to explore the influence of tissue elasticity, rotational velocity, instrument orientation, and tissue shape on the transportation rate, efficiency, and reliability. Tissue phantoms with gelatine concentrations of 3, 9, and 12 wt% were employed to simulate a range of Young’s moduli from 1 to 110 kPa.

**Results:**

The mean transportation rates for these phantoms were 7.75±0.48, 8.43±1.50, and 8.90±0.56 g/min, respectively. Notably, all phantoms were transported successfully. The perfect reliability exhibited underscores the potential of our instrument as an alternative to aspiration catheters. CONCLUSION: This research presents a significant step forward in the field of tissue extraction, offering a promising approach for MIS with enhanced efficiency and reliability.

## Introduction

In a large variety of medical specialities, including cardiology [1], gynaecology [2], neurology [3] and critical care [4], tissues are extracted as a preventative or therapeutic measure [5]. In preventative procedures, (suspicious) tissue is obtained for diagnostic purposes and adequate sample quality is required for accurate analysis. Alternately, unhealthy or obstructive tissue, such as thrombus or tumorous tissue, is removed in therapeutic procedures. Robotic surgery provides safe access to hard-to-reach places to remove tumorous or inflamed tissue [6]. Tissue extraction during surgery is enabled by transportation mechanisms integrated into slender medical instruments, including flexible graspers and aspiration lumens. Medical equipment including bioptomes, stent-retrievers, laparoscopic devices, and catheters all operate using these operating principles [7]. For the elimination of thrombus, for instance, percutaneous aspiration catheters are frequently used. As shorter surgery times are beneficial in order to reduce both anaesthesia time and operation costs [7], continuous transportation mechanisms, such as aspiration, are advantageous over non-continuous methods, such as graspers.

**Fig 1:**
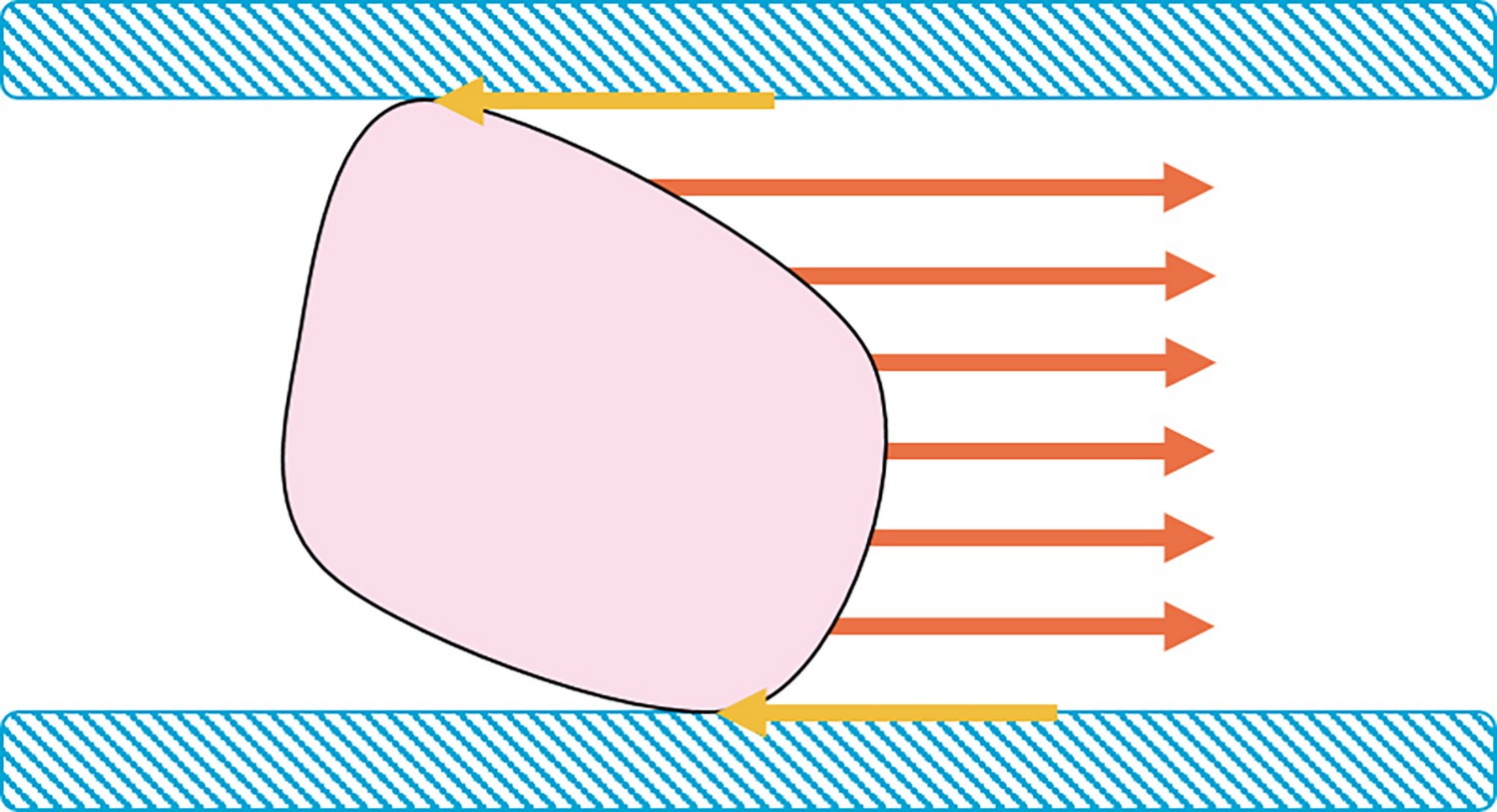
Schematic representation of the forces within an aspiration catheter. The orange lines represent the suction force in the desired direction of translation whilst the yellow lines resemble friction between the transported tissue and the shaft of the device. The effect of gravity is neglected.

Aspiration catheters are currently one of the most frequently utilized medical devices in Minimally Invasive Surgery (MIS) for tissue removal and are consequently referred to as the golden standard [7]. Aspiration catheters consist of a flexible tubular body with a suction channel. The tissue in front of the catheter’s tip is transported through the lumen and towards the handle using a negative pressure differential. Unfortunately, clogging is a common problem with aspiration catheters [7], especially in longer and narrower devices. This phenomenon occurs whenever the friction force between the extracted tissue and the shaft exceeds the suction force (Fig 1). Achieving sufficient pressure differential to transport tissues is challenging due to energy losses that occur over the length of the shaft, which makes them unsuitable for further downsizing or increasing the length [8]. This subsequently also emphasizes the dependency of the reliability of aspiration-based transportation on tissue composition. Stiffer clots are less likely to adjust their shape and, therefore, experience a higher risk of getting clamped within the shaft of the device [9]. Furthermore, the suction force can lead to damage to both the surrounding and transported tissue [7]. The latter is especially a drawback in the case of tissue extraction for diagnostic purposes.

Sakes et al. [7] propose an alternative mechanism for aspiration-based devices, in which tissue is transported using a friction differential between the device and the tissue. Inspired by the egg-laying structure of the wasp, the device consists of six reciprocating semi-cylindrical blades, which move forwards or backwards in a specific motion sequence and enclose the tissue transportation lumen. Assuming that all blades contribute equally to the total friction with the tissue, the tissue will translate in the direction of the majority of the blades. Transportation in the opposite direction can be prevented by limiting the number of blades retreating simultaneously, whilst keeping the remaining blades stationary or moving these forwards. Experimental validation showed that the device is able to reliably transport tissues with an average transportation rate of 0.25 ± 0.04 g/min for tissue with a Young’s modulus of approximately 55 kPa. Using a friction-based transport mechanism will decrease the dependency on tissue elasticity and allow for further downsizing of tissue transportation mechanisms without loss of functionality. However, even though the mechanism shows promising results, the transportation rate of the tissue is still dependent on tissue elasticity, although clogging is no longer an issue [7]. It is hypothesized that soft incoherent tissues potentially deform or shear near the blade surface, resulting in lower transportation efficiencies. Whereas on the other extreme, stiff, incompressible, tissues lack tissue deformation, which results in insufficient contact points between the tissue and reciprocating blades for friction-based extraction. Furthermore, due to the movement sequence, in which at least one blade moves always in the opposite direction of the desired transport direction, the overall transportation rate is relatively low. Instead of using a reciprocating motion, an alternative would be to use a mechanism that solely moves in the desired transport direction, such as a conveying mechanism. This would decrease the dependency on tissue properties and improve the transportation rate.

Based on the challenges of current tissue transportation mechanisms, the main objective of this research is to design a novel continuous tissue transportation mechanism compatible with MIS using friction with the tissue to its advantage and in which the transportation reliability is independent of tissue elasticity. The transportation reliability, transportation velocity and transportation efficiency are measured to compare the transportation performance of the novel tissue transport mechanism with commercial tissue transportation devices. The effects of tissue stiffness, tissue shape, instrument angle and rotational velocity are analysed. With these experiments, we show that the reliability of this novel mechanism remains unaffected by factors such as tissue elasticity, tissue shape and device orientation. Note that the scope of this study does not include the procedure of tissue manipulation and resection.

## Material and methods: Design process

### Compatibility for minimally invasive surgery

Compatibility for MIS relates to multiple aspects of the device such as dimensions, material selection, and design decisions considering safety. All elements can be decisive in whether the instrument can be utilized for tissue removal within the human body and inserted through a small incision. In order to enable insertion through a small incision, we set a maximum outer diameter (∅outer) of 10 mm and a minimum inner diameter (∅inner) of 5 mm. Aspiration catheters’ outer diameters typically range from 3–6 F [10], which corresponds to a diameter of 1–2 mm. However, depending on the type of MIS, other dimensions apply. For example, the average diameter of commercially available colonoscopes is 13 mm [11]. Furthermore, the ability to transport tissue should be independent of the diameter and length of the device, to allow for further downscaling in the future.

In order to enable fast and reliable tissue transport, the device should transport a wide range of tissues. The mechanical properties of tissue both vary amongst different types and change when diseased. For example, Yu et al. [12] found that the Young’s modulus of a tumor within a kidney is approximately 20 kPa higher compared to healthy kidney tissue. Furthermore, strain rates non-linearly influence the measured tissue elasticity [13]. Based on data on the biomechanical properties of soft tissue [13–15], the working range for the to-be-developed transportation mechanism is set to 1–110 kPa. This represents a large range of tissues and organs, from brain tissue and adipose tissue at low strain rates (±1 kPa [13]) to fibrous tissue such as tendons (51.5 ± 25.1 kPa [14]) to cancellous prostate tissue (30–110 kPa [15]), which can be encountered during MIS. Aspiration catheters are assumed to be suitable for the removal of liquids. Therefore, this form of matter is excluded.

In order to enable fast and reliable tissue removal, the transportation rates of the proposed instrument should be comparable to currently clinically available instruments. Transportation rates of commercially available catheters vary between 1.03 and 4.93 mL/s [10]. These velocities are achieved when aspirating liquids and are thus not representative of the removal of solid tissue. More indicative are the transportation rates of clinically available morcellators. These vary between 6.2 to 40.4 g/min during laparoscopic surgery [16]. Therefore we aim to have a similar transportation rate to what is achieved in currently clinically available morcellators.

### Final design

#### Enable cable-driven transport

**Fig 2:**
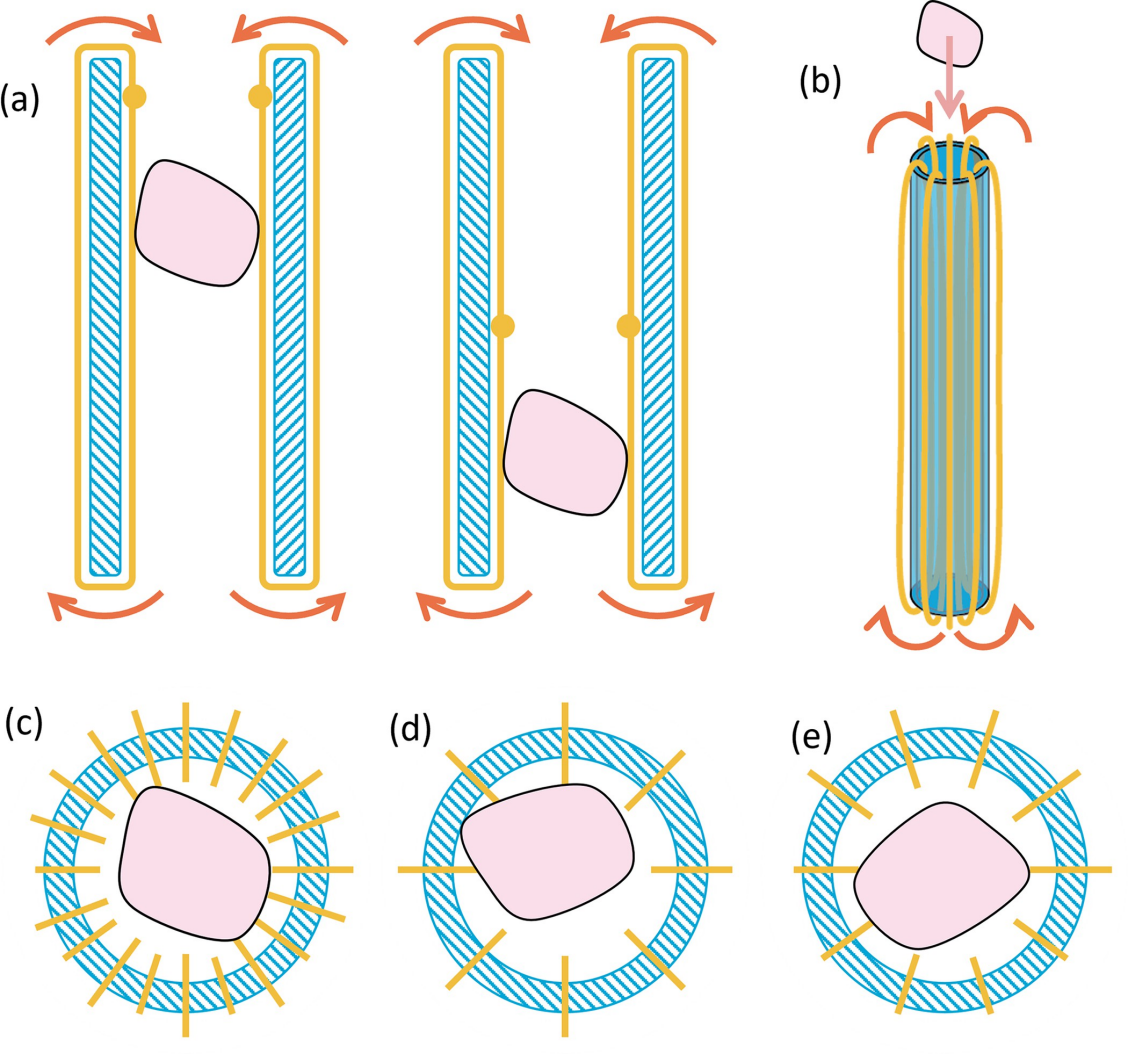
Schematic longitudinal and radial cross-sectional visualisations of the tubular body of the transportation concept. Schematic longitudinal and radial cross-sectional visualisations of the tubular body of the transportation concept. (a) Conveying movement of the wire shown by the yellow dot. (b) Transportation concept in which the continuously rotating cables take the tissue along the tubular body due to the friction between the cables and the tissue. (c) Maximizing points of contact between the cables and piece of tissue requires large actuation force. (d) Minimizing points of contact between the cables and piece of tissue causes risk of sticking to tubular wall, and (e) Final cable design in which one cable is wrapped ten times around the tubular wall. The yellow lines represent the rotating cables and the pink shape the tissue.

The proposed mechanism enables tissue transportation by mimicking the working principle of a conveyor belt. Instead of a rotating planar surface, a cable is spanned along the inner tube of the instrument, which is subsequently actuated to generate a translational motion. Similar to the design of Sakes *et al*. [7], friction between the tissue and the body of the medical instrument is used as an advantage. Due to friction between the cable(s) and tissue, the latter is transported whenever continuously rotating the cable(s) in the direction of removal (Fig 2A and 2B). Effective tissue transportation of this novel mechanism depends on the cable configuration, the cable guiding through the instrument, the friction regulation between the cables and the instrument and efficient actuation of the cables.

**Fig 3:**
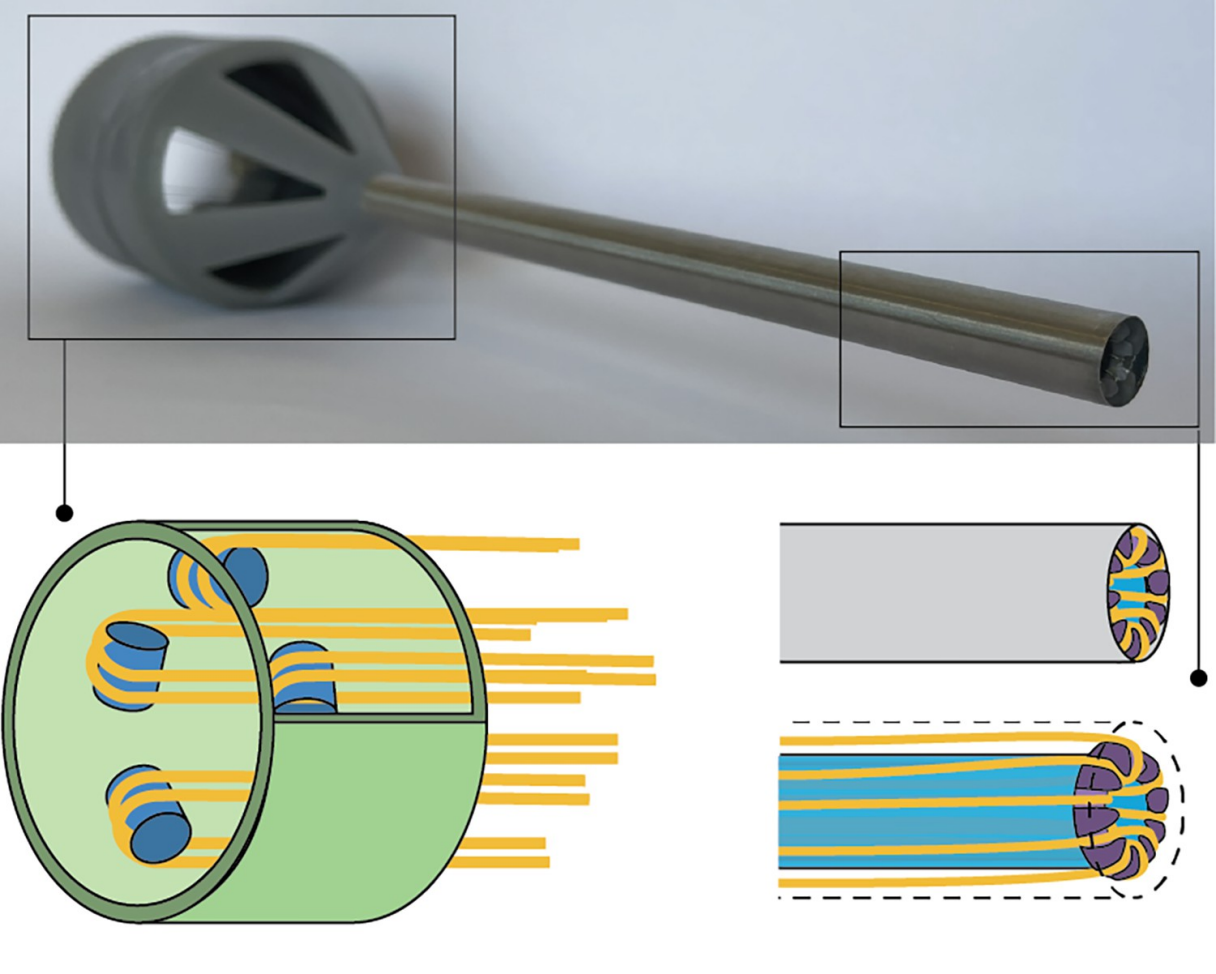
Schematic view of the actuation side of the device and the distal end of the cone. Viewed with and without the outer tube.

Different cable configurations were validated for the suitability of tissue transportation. Increasing the number of loops wrapped around the tube decreases gaps between the cables, further enclosing the transported tissue and maximising the potential points of contact between the tissue and cable, see Fig 2C. The latter is beneficial since friction between the tissue and cable is desirable within the implemented working principle. However, increasing the number of loops of cable simultaneously increases the friction between the cable and the instrument, requiring a larger force for actuation and potentially increasing the chance of wear. As illustrated in Fig 2D, if the device contains too few loops, the tissue is at risk of sticking to the tubular wall. In the final design, it was decided to use one cable wrapped around the tubular body a total of ten times, see Fig 2E. The loops are evenly distributed resulting in a spacing of approximately 1.4 millimetres. Continuous tissue transportation is achieved by clamping each loop of cable along a revolving axis. Whilst it might be favourable to actuate all loops separately, this simultaneously demands more space to position all ten rotating mechanisms. Within the final design, a total of five points of actuation, distributed equally along a circular path was chosen. Fig 3 shows a schematic overview of the cable-driven transport mechanism.

#### Enable low-friction cable movement

**Fig 4:**
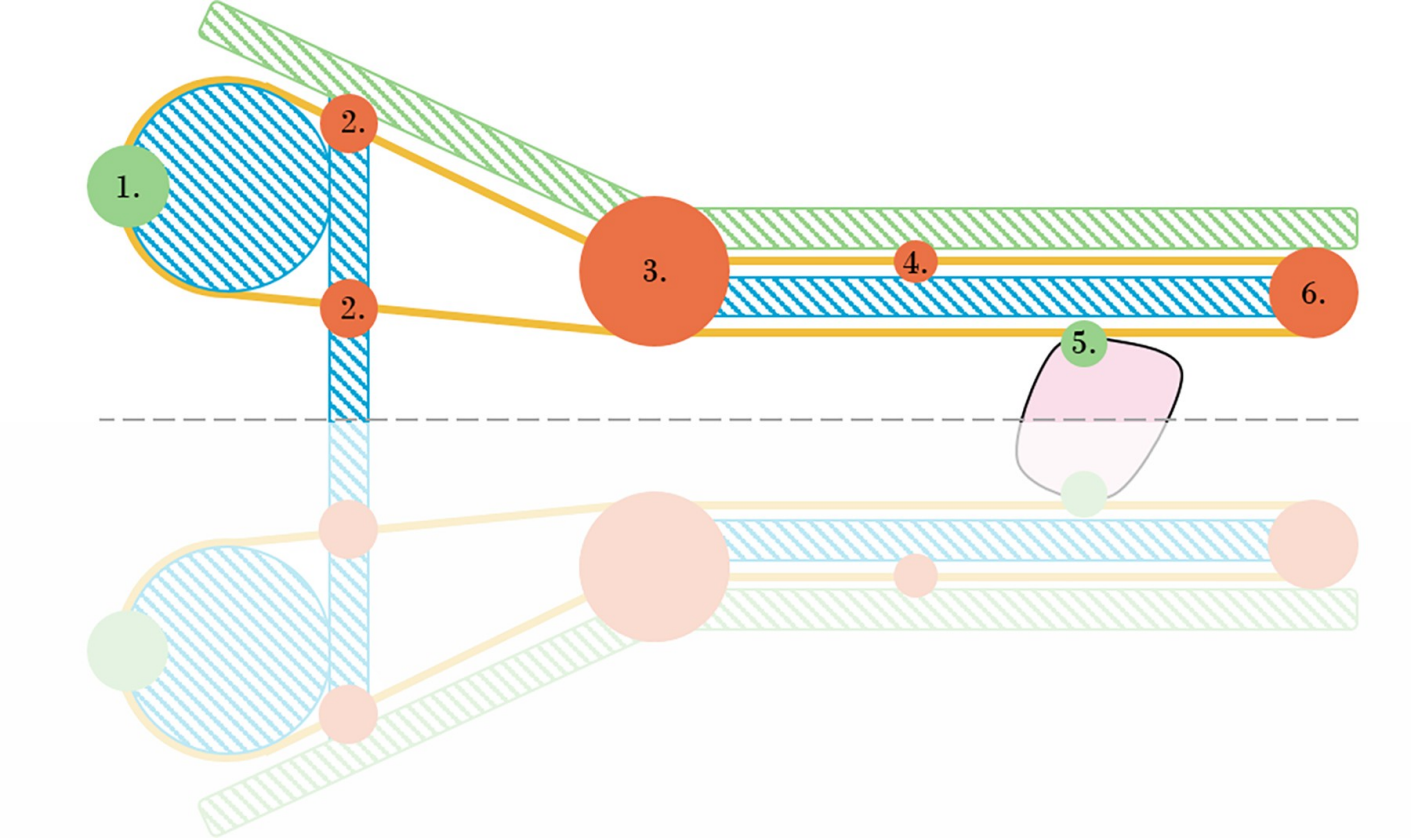
Schematic overview of the device. Green and orange dots resemble areas in which friction is either desired or not, respectively.

Within the designed mechanism, the physical phenomenon of friction is used as an advantage to transport tissue. However, even though high friction is desired between the tissue and cable, there are also regions in which friction is experienced as an obstacle, see Fig 4. Also, note that specific regions of friction within the device are dependent on the amount of cable tension. Namely, whenever the cable is completely stretched, the contact area between the cable and both the tip and actuation axes is increased. Since it is not beneficial for the former but is desired for the latter, a trade-off remains.

High friction between the cable and the shaft of the device is unwanted, as it will result in a higher force that is required to actuate the mechanism, as well as wear. To minimise this hindrance, the cable is spanned with an offset of approximately 0.6 mm with respect to the shaft, as illustrated in Fig 3. However, at the tip of the device, the cable is required to be guided, automatically resulting in a point of contact. In order to prevent kinking and wear of the cable, a grooved tip guides the cable loops through slots resulting in a curvature with a diameter of 1 mm. Furthermore, this tip ensures proper positioning of the cables throughout actuation. Within the handle, the cable is guided over a larger diameter to allow for easy tissue removal and to generate space for the actuation mechanism. The cable is spanned with an offset with respect to the handle body, to avoid undesired friction. At the actuation axes (see Fig 3), however, high friction is needed in order to propel the cable in the specified direction.

#### Prototype development

**Fig 5:**
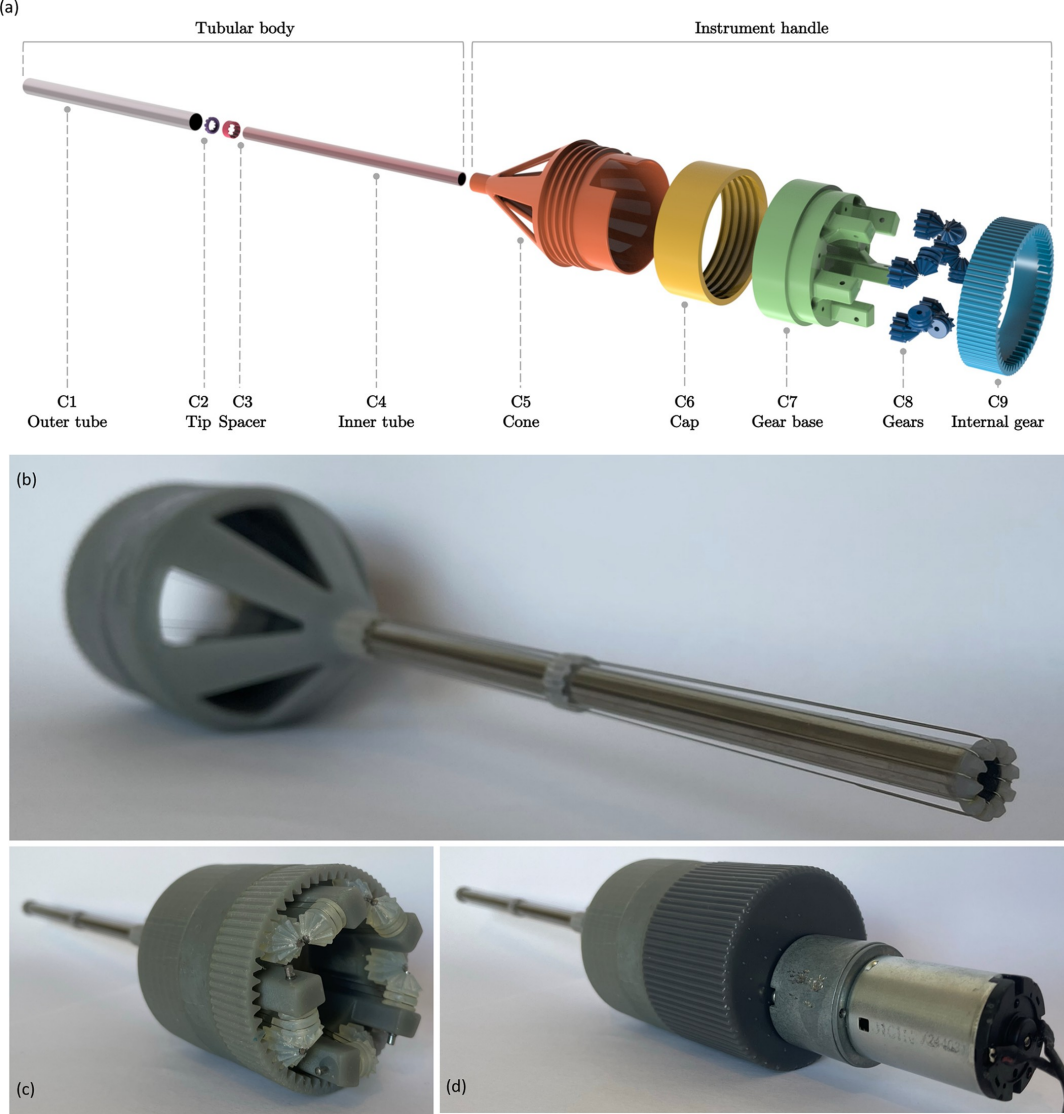
Final design. (a) Exploded view of the proof of principle prototype with its components (C). (b) Device excluding the outer tube. (c) Gearbox of device, and (d) Device configuration for motorized actuation.

**Fig 6:**
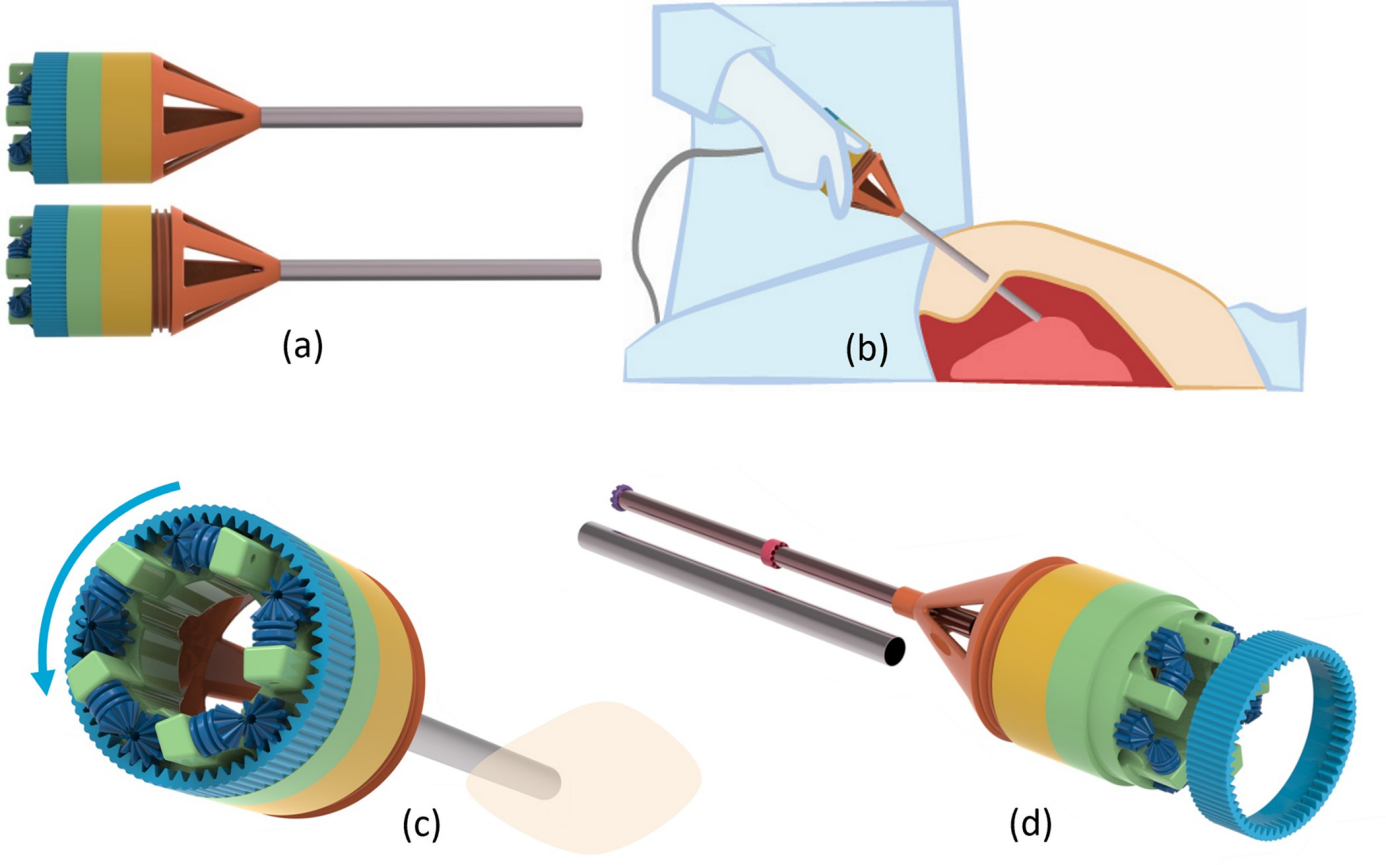
Usage steps. (a) Tensioning of cable, (b) Instrument insertion in the human body through a 10 mm diameter incision, (c) Tissue removal through motorized rotation of the internal gear, and (d) Potential device disassembly for complete or partial re-usage.

The final proof of principle prototype is illustrated in Fig 5. Additionally, a selection of usage steps is visualised in Fig 6. The final prototype consists of 24 parts. All elements, excluding the axes, shafts, and cables, were manufactured through Additive Manufacturing (Form 3B, Formlabs, Somerville, MA, USA). The cone includes the cable tensioning mechanism, form lock, gaps for tissue extrusion, and guiding apertures for all loops of the cable. The cable tensioning mechanism consists of a screw thread that increases the distance between the tubular body and gears, which subsequently tensions the cables.

The design of the actuation mechanism is optimised to enable the passing of the cable, provides sufficient stiffness for the forces on the actuation axes and provides attachment to the cone. Since all five actuation points have a differently orientated axis of rotation, bevel gears were implemented, see Fig 5C. The rotation of the integrated internal gear results in the rotation of all other gears, subsequently rotating all cables simultaneously. In order to increase the friction with the cable, the actuation axes contain two V-slots, both narrowing towards the centre of the axis. Due to this decline in width, the cable is clamped within, and whenever the axis rotates, the cable is pulled in the same direction.

The shaft is made from two Stainless Steel capillary tubes with a length of 150 mm through which the cable runs. The outer tube has an outer diameter of Ø10 mm and a wall thickness of 0.2 mm. The inner tube has an outer diameter of Ø7 mm and a wall thickness of 0.4 mm. The shaft is kept in place using a spacer that also serves as a cable guide and is press-fitted on the cone. At the distal end of the shaft, a 3D-printed tip was press-fitted to maintain cable positioning and prevent kinking. The cable within the final prototype is made from stainless steel and consists of nineteen Ø0.2 mm intertwined smaller cables (1x19). This dimension is selected in a trade-off between minimizing size whilst maintaining sufficient mechanical properties. After wrapping the cable around the shaft, both ends were connected to each other using a flat knot.

## Material and methods: Experimental validation

### Experimental goal

The main goal of the experiments is to determine the transport reliability of the designed transportation mechanism for a range of rotational velocities of the internal gear and the elasticity of the tissue (Experiments 1–9), the angle at which the device is operated (Experiments 10–12), and sample shape of the transported tissue (Experiment 13). The secondary outcomes of the experiments are the transportation velocity and the transportation efficiency, such that the performance of the developed transportation mechanism can be compared to currently clinically available tissue transport devices.

The independent variables are:

- Tissue elasticity. The transportation mechanism ought to be suitable for tissues within the elasticity range of 1–110 kPa. This represents a large range of tissues and organs which can be encountered during MIS. To mimic the mechanical characteristics of these tissues, tissue phantoms were manufactured using a gelatine-water mixture. The implemented samples have a gelatine weight percentage (wt%) of 3, 9, and 12, representing a tissue elasticity of approximately 1–10, 55, and 100–110 kPa, respectively [17]. Red food colouring was added to enhance the visibility of the tissue phantoms.
- Rotational velocity. The developed mechanism is actuated by the rotation of the internal gear. Increasing the velocity of this motion subsequently increases the translation rate of the cables and, therefore, the tissue transportation velocity. However, it might be hypothesised that high velocities of the cable can potentially result in slip with the tissue. Therefore, this aspect is varied within the experiments to indicate its influence. To guarantee consistent rotation of the proof of principle prototype, actuation is facilitated through an electromotor. Experiments are executed with three different actuation velocities, resulting in a voltage of 8.5, 11, and 13.5 V. The lower extremity is the minimum required voltage in order to set the internal gear in motion. The subsequent rotational velocity is expressed in Rotations Per Minute (RPM). Note that identical power supply voltages do not directly imply equivalent rotational velocities. This is due to different friction forces between the cable and instrument between the experiments as a result of varying cable tension. Therefore, no absolute values can be retrieved from the comparison between tissue elasticity groups, the relative outcomes are merely indicative.
- Instrument orientation. Within MIS, the tubular body of the device is advanced through the human body until correctly positioned for removal. In order to access these target locations, the orientation of the instrument is varied gradually, which impacts the effect of gravity. For example, a more upright position of the tubular body with the tip pointed downwards results in a force on the tissue opposite to the direction of removal. Whenever the gravitational force is higher than the friction between the cable and tissue, removal is obstructed. Within the experimental setup, the operation of the proof of principle prototype is tested horizontally and by pointing the tip of the instrument downwards at an angle of 20°.
- Tissue phantom shape. Transportation within the designed mechanism is enabled through friction between the cable and tissue. These loops of cable are evenly distributed along a circular path, subsequently spanning a tubular body. Whenever this body is completely filled with tissue, all cables contribute to the transportation of the tissue, maximising the positively experienced friction between both. Whenever this is not the case, fewer cables affect this motion. To map this influence, tests are executed with cylindrical and half-cylindrical tissue phantoms. The diameter and height of each sample are 5 and 15 mm, respectively.

The dependent variables are:

- Transportation velocity. All previously mentioned independent variables potentially influence the transportation velocity of the tissue phantoms. Minimising the required time for removal is desired within MIS, reducing anaesthesia time and operational costs [7]. Therefore, it is desired to maximise this variable, matching or exceeding the flow rates of currently used devices. The transportation velocity is expressed in [mm/s], calculated by dividing the travelled distance of the tissue phantom within the tubular body by the required time. Additionally, to enable comparison to commercially available devices, an estimation is made of the corresponding transportation rate in [g/min], calculated by dividing the average tissue phantom weight by the average time required for the concerning to travel through the tubular body.
- Transportation efficiency. Transportation efficiency comprises whether the travelled distance of the cables corresponds with the translation of the tissue. Within an ideal scenario, these values are identical. However, whenever friction between the cable and tissue is insufficient, slip occurs, decreasing the translation of the latter with respect to the former. This can for example be caused by inadequate contact points between both, due to larger experienced friction between the tissue and tubular wall of the device, or due to gravity forces adverse to the direction of removal. Since *time* [s] is measured within the experiments, the transportation efficiency is calculated as indicated in Eq (1) and Eq (2). Within this equation, *η*_*transportation*_ represents the transportation efficiency in [%] and *t*_*theoretical*_ [s] and *t*_*measured*_ [s] the theoretical and measured translation time, respectively. The rotational velocity of the motor, *RPM*_*motor*_, is converted to the corresponding rotational velocity of the spur and bevel gears, by multiplying this variable with the teeth ratio of the internal and spur gear, *teeth*_*internal*_ and *teeth*_*spur*_, respectively. The fraction of the travelled tissue distance, *∆x*_*tissue*_ [mm], divided by the circumference of the pulley directly attached to the bevel gear, *c*_*pulley*_ [mm], determines the theoretically required number of rotations for this translation. Dividing the second element by the first and multiplying with a factor of sixty results in the theoretical translation time. Note that a perfect transmission between gears and between the clamping axes and cable is assumed.

$$\eta_{transportation=\frac{t_{theoretical}}{t_{measured}}∙100\%}$$ (1)

$$t_{theoretical}=\frac{\frac{{\Delta x}_{tissue}}{c_{pulley}}}{{RPM}_{motor}∙\frac{{teeth}_{internal}}{{teeth}_{spur}}}∙60$$ (2)

- Transportation reliability. Lastly, the variable transportation reliability indicates whether all tissue phantoms are successfully transported within the executed experiments. Phenomena such as clogging or malfunctioning of the device are unacceptable within medical procedures. The transportation reliability *r*, expressed as a percentage [%], is calculated as indicated within Eq (3). *n*_*successful*_ represents the number of times in which the tissue phantom is successfully transported through the tubular body of the device and *n*_*total*_ is the total amount of experiments executed. This variable is aimed to be as high as possible.

$$r=\frac{n_{successful}}{n_{total}}∙100\%$$ (3)

### Experimental procedure

**Fig 7:**
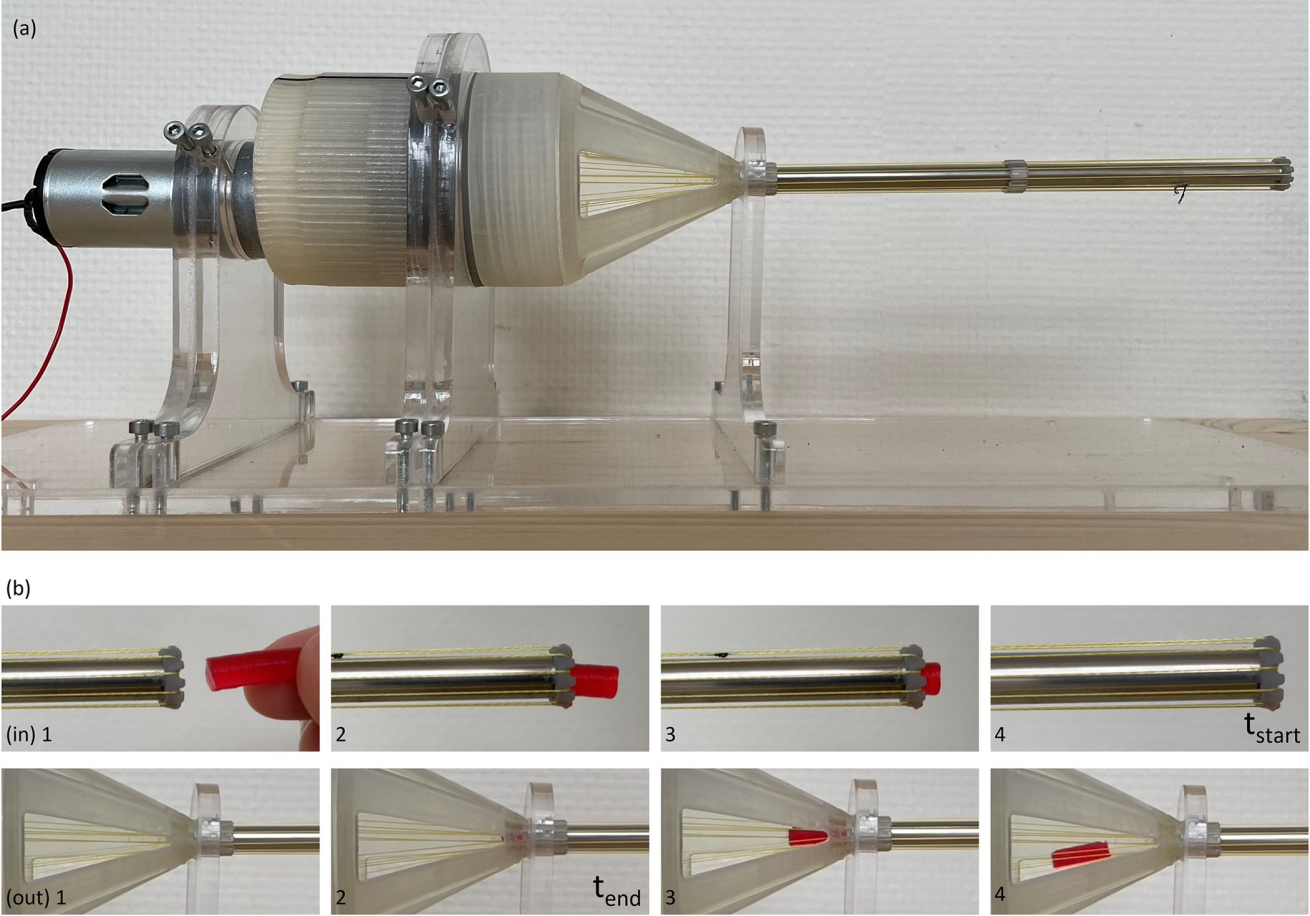
Experimental setup. (a) Proof of principle prototype mounted in a PMMA standard. (b) Snapshots of a tissue phantom entering and exiting the tubular body of the device, indicating the configuration in which the start and end times are determined, used to calculate the transportation velocity.

The experimental setup is illustrated in Fig 7A. The transport system is mounted in a PMMA standard and supported at three fixed locations. One of the five holes within the cone is orientated perfectly towards the point of vision, allowing for clear detection of the transported tissue phantoms. Behind the gearbox, an electromotor (Igarashi 33GN2738-132-GV-5 50:1, 12V) is placed to actuate the prototype. This is facilitated by attaching the axis of the electromotor by means of a shape lock to the internal gear. A power supply is attached to the electromotor. Furthermore, a black line was drawn on the outside of the internal gear to determine the rotational velocity of the prototype, which is expressed in RPM. Also, the knot used to tie both ends of the cable to one another was marked black and was used as a reference to determine the rotational velocity of the cable. The outer tube is removed throughout the experiments. To ensure identical tissue phantom sample sizes, the different gelatine mixtures were poured into moulds, containing cylindrical cut-outs with a diameter of 5 mm and height of 15 mm. The half-cylindrical tissue phantoms were cut manually by splitting the cylindrical shapes in half.

In total, 13 experiments were carried out, see Table 1. Each individual experiment was performed with ten tissue phantoms. The prototype was mounted in the experimental setup, after which the electromotor was turned on. Subsequently, the tissue phantom was inserted manually at the tip of the device. Once the tissue phantom travelled the entire distance through the tubular body and was visible within the handle, the measurement was terminated. After this, the process was repeated with a new tissue phantom for a total of ten times. Each experiment was recorded using a camera.

**Table 1:** Overview of the executed experiments using the proof of principle prototype. Each experiment was executed with ten tissue phantoms, resulting in a total of 130 measurements.

Experiment number	1	2	3	4	5	6	7	8	9	10	11	12	13
Power supply (V)	8.5	8.5	8.5	11	11	11	13.5	13.5	13.5	11	11	11	11
Tissue phantom (wt%)	12	9	3	12	9	3	12	9	3	12	9	3	9
Device orientation (°)	0	0	0	0	0	0	0	0	0	-20	-20	-20	0
Tissue Phantom Shape	cylinder	cylinder	cylinder	cylinder	cylinder	cylinder	cylinder	cylinder	cylinder	cylinder	cylinder	cylinder	½ cylinder
Transportation Efficiency (%)	91	91	47	100	91	44	95	89	90	92	84	75	91
Reliability (%)	100	100	100	100	100	100	100	100	100	100	100	100	100

### Data analysis

The video footage obtained during the experiments is evaluated manually by systematically freezing the video frames to extract the RPM of the internal gear and the required time for each tissue phantom to travel through the tubular body of the device. Within Fig 7B, several snapshots are displayed of a tissue phantom entering and exiting the tubular body. The rotational velocity of the cable is approximated by counting the number of times the black line on the outside of the internal gear passes by throughout all ten measurements, divided by the total experiment time. The retrieved information is processed within Matlab, whereby Boxplots and statistical analysis are utilised to gain insights. One-way ANOVA and independent two-sample t-tests are performed for data analysis.

## Results

**Fig 8:**
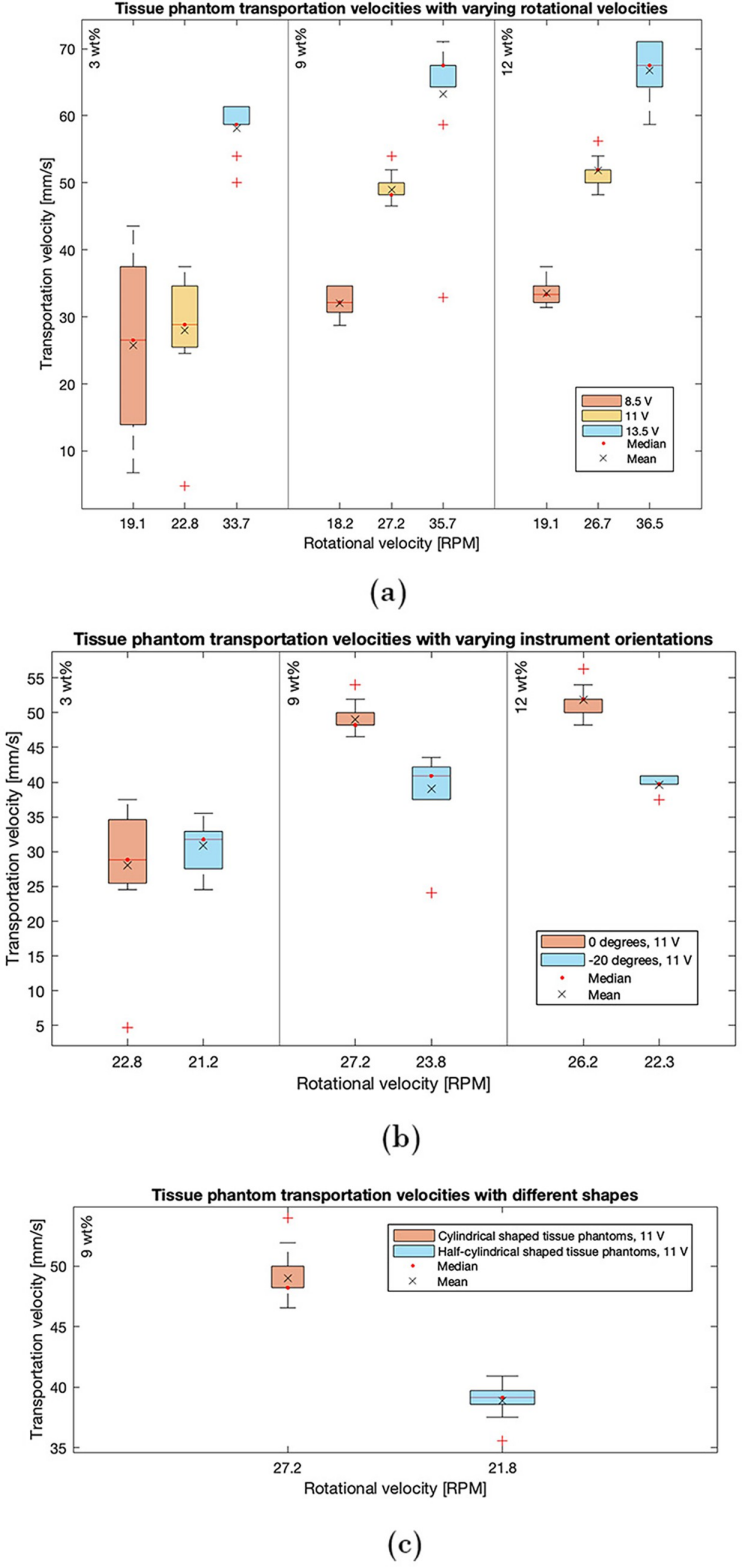
Results illustrated in boxplot graphs. Outliers are plotted individually using the red ‘+’ marker symbol. (a) The transportation velocities of all three weight percentage gelatine tissue phantom categories as a result of varying rotational velocities. (b) The transportation velocities of all three weight-percentage gelatine tissue phantom categories as a result of varying instrument orientations, all actuated with a power supply of 11 V. (c) The transportation velocities of all three weight-percentage gelatine tissue phantom categories as a result of varying tissue phantom shapes, all actuated with a power supply of 11 V.

The data extracted from Experiments 1–9 (see Table 1) is displayed in Fig 8A. Within these experiments, the goal was to gain insights into the dependency of the transportation velocities of tissue phantoms with differing elasticity properties on the rotational velocity of actuation. It can be seen that an increase in rotational velocity subsequently increases the transportation rates for all three categories. A statistically significant difference in the transportation velocity caused by deviating power supply is established through one-way ANOVA analysis, for the 3 wt% (F (2, 27) = 33.13, p = 5.40 10^−8^), 9 wt% (F (2, 27) = 53.55, p = 4.01 10^−10^) and 12 wt% (F (2, 27) = 316.82, p = 1.80 10^−19^) gelatine tissue phantoms. With the current set-up, the highest transportation rates are attained through a 13.5 V power supply for all three groups, resulting in a mean velocity of 58.16 ± 3.59 mm/s, 63.23 ± 11.24 mm/s, and 66.75 ± 4.19 mm/s for the 3, 9, and 12 wt% gelatine tissue phantoms, respectively. This resembles a mean transportation rate of 7.75 ± 0.48 g/min, 8.43 ± 1.50 g/min, and 8.90 ± 0.56 g/min accordingly, assuming a tissue phantom mass of 0.3 gram. The highest variance is measured whilst transporting 3 wt% gelatine tissue phantoms with a power supply of 8.5 V, resulting in a mean velocity of 25.80 ± 13.88 mm/s.

Within Fig 8B, data extracted from Experiments 4–6 and 10–12 (see Table 1) are graphically displayed. In these experiments, the transportation velocity is measured with varying instrument orientations for all three weight percentage gelatine tissue phantoms. For the 9 and 12 wt% gelatine tissue phantom categories, higher transportation velocities were attained whenever the device was oriented horizontally when compared to the tip pointing downwards at an angle of 20°. This is confirmed by the independent two-sample t-tests, indicating significantly different transportation velocities for the 9 (h = 1, p = 5.38 10^−4^) and 12 wt% (h = 1, p = 3.47 10^−7^) gelatine tissue phantoms. Within the 3 wt% gelatine phantoms, no significant difference is found between the transportation velocities within varying instrument orientations according to an independent two-sample t-test (h = 0, p = 0.3511). Lastly, different shapes of tissue phantoms were transported within the proof of principle prototype. This experiment was only executed for 9 wt% gelatine tissue phantoms with a corresponding power supply of 11 V. The results are graphically summarized in Fig 8C. Both the half-cylindrical and cylindrical tissue phantoms were reliably transported with a transportation rate of 100%.

The transportation efficiency of the device was determined, as indicated by Eq 1. The results, based on the mean tissue transportation time and rotational velocity of the internal gear of each experiment, are displayed in Table 1. It was found that the transportation efficiency decreases whenever the tip is pointed downwards at an angle of 20° with the 9 and 12 wt% gelatine tissue phantoms. Efficiency remains identical in the case where the tissue phantoms are halved and thus adjusted in shape. In Experiments 3 and 6, efficiency is significantly lower at 47% and 44%, respectively.

## Discussion

### Summary of main findings

The proof of principle prototype enables continuous transportation of tissue within a Young’s modulus range of 1–110 kPa, mimicked using 3, 9, and 12 wt% gelatine tissue phantoms with a transportation reliability of 100%. The maximum mean mass transportation rate of these categories is measured to be 7.75 ± 0.48, 8.43 ± 1.50, and 8.90 ± 0.56 g/min, respectively. This lies within the range of transportation rates of clinically available morcellators operated during laparoscopic surgery, such as the Steiner morcellator (Karl Storz). This morcellator has a mean transportation rate of 6.3 g/min [16]. However, laparoscopic morcellators such as the Gynecare Morcellex (Ethicon) reach higher transportation rates of around 28 g/min [18]. Similar transportation rates could potentially be reached by an increase in the rotational velocity of the developed transport mechanism, as the experiments showed that this significantly increases transportation rates for all three phantom tissues. During the experiments, no stagnation and corresponding equilibrium were retrieved which means that the maximum transportation velocity of the developed transportation mechanism was not reached yet. Transportation efficiency increases for tissue phantoms with higher elasticity properties. It is hypothesized that soft tissues are more likely to deform or shear near the cable surface, being more sensitive to slip. At the other extreme, stiffer tissues clamp within the cable loops and therefore experience high friction between both, which is beneficial for the developed transportation mechanism. Concerning shaft orientation, an efficiency loss of approximately 8 and 7% was found when pointing the tip of the device downwards when compared to horizontal operation, for transportation of the 9 and 12 wt% gelatine tissue phantoms, respectively. Efficiency remained identical in the case where the tissue phantoms were halved, and thus adjusted in shape. It is expected that the decrease in tissue phantom volume and weight measures up to the decrease in contact points between the concerning and cable. Alternatively, it can be hypothesized that not all contact points are required for friction-based transport of the tissue. The mechanism’s independence on tissue volume is, for instance, beneficial for the removal of tissue during laser surgery, which is associated with variable shrinkage of the tissue specimen [19]. With the current advancements in exoscope-based surgery, it becomes even more evident to remove irregular tissue from hard-to-reach surgical sides [20]. Throughout the experiments, all tissue phantoms were transported successfully through the tubular body of the device, resulting in a reliability of 100%. The developed conveying transport mechanism outperforms other tissue transport devices, such as aspiration-based devices or the friction-based transportation device developed by Sakes et al. [7], in the ability to transport tissue independently of their elasticity or shape. Moreover, unlike aspiration-based devices, surrounding tissue is unaffected by the transportation mechanism and the conveying transportation principle allows for miniaturization without compromising on performance.

### Limitations

The proof of principle prototype showcased the working principle of using a conveying mechanism for tissue transportation. However, as expected, undesired friction occurs between the spanned cable and the tip of the instrument, resulting in excessive wear. Subsequently, the bending radius of the cable decreased, which increased the experienced friction. Furthermore, whenever the tissue reached the proximal end of the tubular body, the translation velocity of the tissue rapidly decreased due to the gradual loss in contact area with the cable. Due to this occurrence, the tissue often remained partially trapped within the tubular body. Although this might not be an issue during continuous operation, a slight redesign of the cable guidance in the handle can help prevent this in future. Additionally, the rotational velocity of the actuation is varied between experiments by altering the power supply of the electromotor, expressed in voltage. Dependent on the, at that time, experienced resistance, this resulted in a specific rotational velocity of the internal gear, which is thus not identical within each experiment powered with the same voltage. Finally, future research should include *ex-vivo* and *in-vivo* studies to gather more insight into the performance of the developed tissue transportation mechanism. Although the gelatine phantom tissues mimic the stiffness of a wide range of tissues, these phantom tissues are unable to fully resemble all tissue properties accurately, such as their heterogeneous composition and friction coefficient. These properties could affect the interplay between the transported tissues and cables.

### Recommendations

In future, the device should be redesigned to tailor the desired friction to its function and prevent excessive wear. More rubber-like materials in the gears might be advantageous to enhance the clamping functionality, whilst a wear-resistive material could be applied for the bevel gears. To prevent excessive wear of the tip due to cable sliding, it is recommended to make use of an alternative material, such as stainless steel, for this component. Excessive cable tension resulted in slight bending of the gear base and play between the electromotor and the housing caused a slightly jolty motion of the device. In order to prevent play between the components, the gear base and electromotor connection should be fortified and redesigned, for instance by supporting the bevel gear’s axes at both sides to ensure the alignment of the bevel gears. With these adaptations, the durability of the transportation device should be demonstrated in fatigue studies. In order to increase access to different clinical application areas of the device, the shaft could be made flexible or steerable to enable the circumnavigation of delicate structures and bones. However, when positioning the device within a curvature, a length difference occurs between paths along the tube. To maintain tension along all loops of cable, the corresponding tensioning mechanism and cable implementation ought to be adjusted. Additionally, the ability of the device to grip tissues could be investigated, as only a small area of contact resulted in the device pulling the tissue into the shaft. The developed transportation mechanism could be integrated into existing devices used in, for instance, Natural Orifice Transluminal Endoscopic Surgery (NOTES), endoluminal surgery or laparoscopic surgery. The inner lumen provides space for the insertion of other instruments used during these procedures, such as scalpels and graspers, or imaging tools, such as an endoscope. Integrating such instruments with the transportation mechanism eliminates the need for additional incisions. Also, future studies should validate the performance of the device in *ex-vivo* and *in-vivo* (animal) studies and should encompass a wide range of tissues, such as uterine, bowel or muscle tissue, featuring different tissue shapes and volumes. This would not only provide valuable insights into the mechanism’s versatility, but the transported tissue samples can additionally be analysed for superficial and histological damage. Damage could be caused by tissue heating (due to friction forces) or tissue fragmentation (due to shear forces generated by the cables). Lastly, future research should focus on sterilizability to make the device applicable for clinical use.

## Conclusion

The goal of this study was to design a continuous tissue transportation mechanism compatible with MIS in which the transportation reliability is independent of tissue elasticity. The designed instrument enables tissue removal using friction between a continuously rotating conveying cables mechanism. The proof of principle prototype demonstrated the feasibility of the mechanism in successfully transporting 3, 9, and 12 wt% gelatine tissue phantoms, resulting in the reliability of the instrument of 100%. The maximum mean mass transportation rate of these categories was measured to be 7.75 ± 0.48, 8.43 ± 1.50, and 8.90 ± 0.56 g/min, respectively, illustrating the achieved transportation velocities lay within the rates of clinically available morcellator operated during laparoscopic surgery. Furthermore, potential barriers of the currently golden standard aspiration catheter are overcome, such as the risk of clogging, damage hazard to surrounding tissue, and the dependency of the force of removal on the diameter of the tubular body. All these elements contribute to the potency of the designed mechanism to become a viable tissue transportation mechanism for MIS in future.

## Supporting information

S1 Data Data experiments. (XLSX)
